# Spatiotemporal dynamics and functional characteristics of the composition of the main fungal taxa in the root microhabitat of *Calanthe sieboldii* (Orchidaceae)

**DOI:** 10.1186/s12870-022-03940-y

**Published:** 2022-12-02

**Authors:** Min Huang, Dazhong Gao, Lele Lin, Shengcai Wang, Shaohua Xing

**Affiliations:** 1 School of Ecology and Nature Conservation, Beijing Forestry University, Haidian, Beijing, 100083 China; 2 Ecology and Nature Conservation Institute, Chinese Academy of Forestry, Haidian, Beijing, 100091 China

**Keywords:** Orchidaceae, Endophytic fungi, Soil Fungi, Spatiotemporal dynamics, Fungal ecological functions

## Abstract

**Background:**

Endophytic fungi play a critical ecological role in the growth and development of orchids, but little is known about the spatial and temporal dynamics of fungal diversity or the ecological functions of fungi during orchid growth and reproduction. *Calanthe sieboldii* Decne. is listed in the Chinese National Key Protected Wild Plants as a class I protected wild plant. To understand the community characteristics of root and soil fungi of the orchid during its reproductive seasons, we investigated the community composition, spatial and temporal dynamics, and functional characteristics of the orchid microhabitat fungi by using diversity and ecological functional analyses.

**Results:**

We discovered that there were three, seven, and four dominant fungal families in the orchid's roots, rhizoplane soil, and rhizosphere soil, respectively. Tulasnellaceae, Aspergillaceae, and Tricholomataceae were the dominant fungi in this endangered orchid's microhabitats. The closer the fungal community was to the orchid, the more stable and the less likely the community composition to change significantly over time. The fungal communities of this orchid's roots and rhizoplane soil varied seasonally, while those of the rhizosphere soil varied interannually. Saprophytic fungi were the most abundant in the orchid's fungal community, and the closer the distance to the orchid, the more symbiotic fungi were present.

**Conclusions:**

The fungi in different parts of the root microhabitat of *C. sieboldii* showed different spatiotemporal dynamic patterns. The fungal community near the orchid roots was relatively stable and displayed seasonal variation, while the community further away from the roots showed greater variation. In addition, compared with the soil fungi, the dominant endophytic fungi were more stable, and these may be key fungi influencing orchid growth and development. Our study on the spatiotemporal dynamics and functions of fungi provides a basis for the comprehensive understanding and utilization of orchid endophytic fungi.

## Background

The plants in the family Orchidaceae have high aesthetic value and cultural significance in traditional Chinese culture; therefore, they are frequently stolen and poached, and many species are facing extinction [1, 2]. The Chinese Ministry of Agriculture and Rural Affairs and the National Forestry and Grassland Administration officially released a new version of "The List of National Key Protected Wild Plants" [3] on September 7, 2021, that includes a variety of orchids. *Calanthe sieboldii* Decne. is listed as a class I national protected wild plant, growing in mountain forests at an altitude of 400–1500m [4]. Its flowers are attractive, and its ornamental value is much higher than the ornamental and economic values of the other 12 orchid species in the same genus [5]. *C. sieboldii* is primarily native to Asia and is distributed in Hunan and Anhui provinces in mainland China [6]. This species has a population of less than 1,000 plants according to a field survey done from 2019 to 2022 in Anhui, China, the region with largest number of plants.

Orchid seeds are numerous, but their germination depends on their endophytic fungal partners due to their small size and lack of endosperm [7, 8]. Endophytic fungi that grow inside orchid tissues can be divided into orchid mycorrhizal fungi (OMF) and orchid non-mycorrhizal fungi (ONF) [9]. Only OMF can produce specific pelotons to be digested by the orchids [10], which in turn contributes to orchid seed germination and growth. ONF do not have pelotons in orchid roots or other tissues and do not harm the health of the host plant [11]. Although *C. sieboldii* benefits from the aseptic seed germination technology to obtain a large number of seedlings [12], its subsequent growth would be retarded in the absence of its fungal partners. These aseptic seedlings often have some problems such as low seed germination rates, slow or non-development, and difficult seedling survival [13–16]. Endophytic fungi, in contrast, can boost seed germination, seedling transplanting survival, flower size and number, fresh weight, and plant quality [17–19]. As a result, a thorough description of the endophytic fungal community is essential for this species' conservation.

It is worth noting that the composition of orchid microhabitat fungal communities, including endophytic fungi, is unstable and can change seasonally or interannually [20, 21]. Only a few studies have considered the seasonal dynamics of fungal colonization and community composition in adult orchid roots [22, 23]. For example, *Tulasnella* were found more frequently as orchid mycorrhizal fungi in the summer, and *Leohumicola sp.* dominated in the autumn in the roots of *Pseudorchis albida* L. [24]. Several studies on the temporal dynamics of plant mycorrhizal fungi have been conducted, including studies on arbutoid mycorrhiza and ectomycorrhiza [25–29]. However, little is known about the temporal variation in endophytic fungal diversity during orchid reproductive growth.

Among soil microorganisms, the spatial heterogeneity of fungi is greater than that of bacteria [30]. Nevertheless, the majority of studies on the spatial heterogeneity of fungi concentrate on larger geographical scales such as location (kilometer level) [31], elevation (vertical space) [32], and habitat [33]. The changes in fungal communities at small spatial scales such as ecological niches [34] have received less attention. McCormick et al. and Waud et al. collected soil at various distance gradients at the meter and decimeter levels and discovered that the closer the soil to the orchids, the greater the number of OMF [35, 36]. In this study, we attempted to determine whether a similar pattern exists at a smaller spatial scale (centimeter level) by dividing the orchid root microhabitats into three zones: roots, rhizoplane, and rhizosphere.

In this study, we investigated the temporal variation of plant root, rhizoplane, and rhizosphere fungal communities of *C. sieboldii* using internal transcribed spacer (ITS) metabarcoding and diversity analysis methods. Our main objectives were to answer the following questions: (1) What are the dominant fungi and their ecological functions in the microhabitat of *C. sieboldii*? (2) What are the temporal dynamics of the dominant endophytic fungi in the roots of *C. sieboldii*? (3) Are there differences between the fungal communities of *C. sieboldii* in different phenological periods, years, and spatial categories? Our hope is to provide a theoretical foundation for research aimed at using endophytic fungi to assist this endangered orchid species' growth, reproduction, and conservation.

## Results

### Distribution of dominant fungi in roots, rhizoplane soil, and rhizosphere soil during different reproductive seasons

**Fig. 1:**
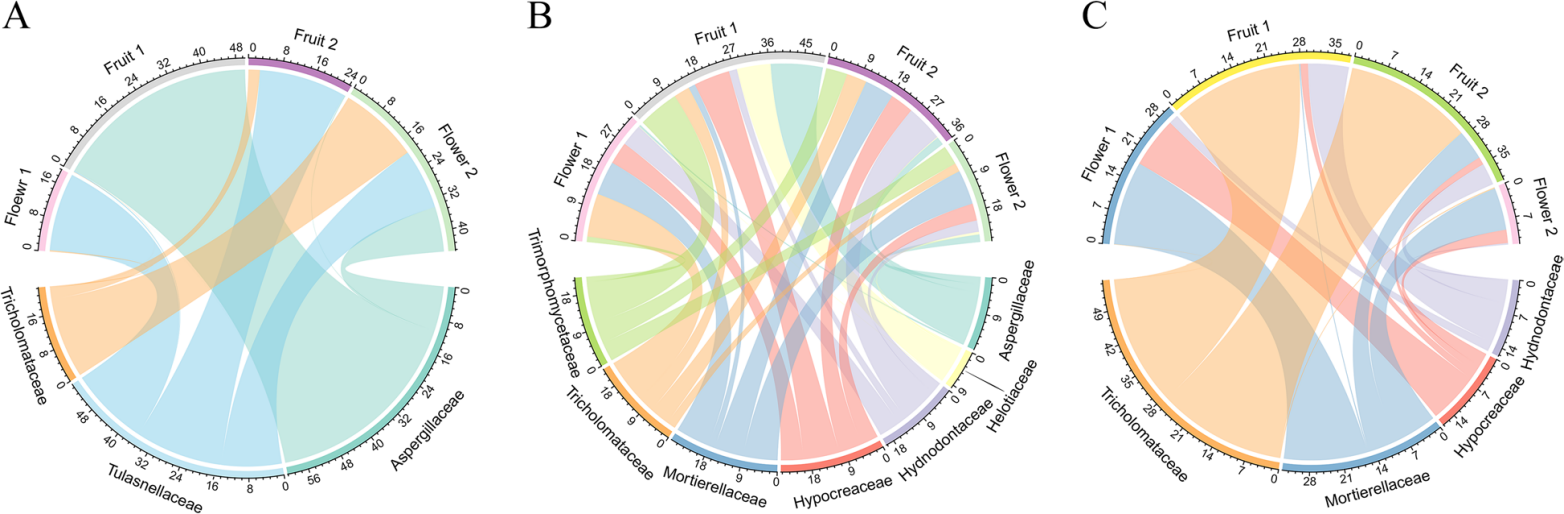
Circos diagrams of dominant fungi at the family level (> 8%). **A** Dominant fungal families in orchid roots. **B** Dominant fungal families in rhizoplane soil. **C** Dominant fungal families in rhizosphere soil. Flower 1, Fruit 1, Flower 2, and Fruit 2 indicate the flowering of 2019, fruiting of 2019, flowering of 2021, and fruiting of 2020, respectively

We collected a total of 12 root samples, 24 microhabitat soil samples, and three control soils from *C. sieboldii* in four phenological periods over three years. After processing, we finally obtained a dataset of 3,422,216 reads with 18,026 ASVs (Table 1). In the roots, rhizoplane soil, and rhizosphere soil we found three, seven, and four dominant fungal families, respectively, and Tricholomataceae was the dominant family in all three spatial categories (Fig. 1). As the dominant fungi in the roots of fruiting plants in 2019, Aspergillaceae had the highest relative abundance, up to 49.77%. Tulasnellaceae was the dominant endophytic fungi in all the periods except for the fruiting in 2019 when it was relatively abundant in the roots of the orchids, indicating that Tulasnellaceae's equilibrium is relatively stable. In addition, Tricholomataceae (19.48%), Tulasnellaceae (14.64%), and Aspergillaceae (10.91%) were the most prevalent fungi in the roots of flowering plants in 2021.

**Table 1:** Fungal sequences of *C. sieboldii*

Group	Root				Rhizosphere soil				Rhizoplane soil				Control soil
	2019	2019	2020	2021	2019	2019	2020	2021	2019	2019	2020	2021	
	Flower	Fruit	Fruit	Flower	Flower	Fruit	Fruit	Flower	Flower	Fruit	Fruit	Flower	
Ave. Sequence reads/ sample	55,154	72,281	73,749	72,719	45,674	63,191	70,724	68,665	57,333	58,927	68,808	68,459	72,123
Number of ASVs	337	1293	1626	4173	970	988	1451	1610	1069	459	1193	1496	1361
% of unclassified	67.27	13.77	40.43	15.85	12.52	8.51	9.98	8.95	7.83	38.23	10.61	13.97	14.57
Number of families	65	140	155	231	111	109	163	176	116	58	141	170	172
Number of genera	87	251	304	511	168	175	292	345	187	87	247	326	326
Sequence length (bp)	207–399				165–312

The root-associated soil was dominated by Tricholomataceae and Mortierellaceae, while the dominant fungi in the control soil were Archaeorhizomycetaceae (19.17%) and Bulleribasidiaceae (15.07%). This demonstrated that the dominant fungi in the microhabitat of the root-associated soil were less associated with the dominant fungi of the control soil, whereas the endophytic fungi of orchid roots and soil fungi of the root-associated soil were distinct, although they still shared two of the same fungal families. As a result, the differences between control soil and root-associated soil were greater than the differences between the fungi of orchid roots and root-associated soil. In the fruiting of 2019, the dominant fungi in the rhizoplane soil were the most abundant among all of the samples, with a total of four families. These families were Aspergillaceae (13.39%), Trichorphomycetaceae (10.51%), Hypocreaceae (9.3%), and Helotiaceae (8.7%). Furthermore, the rhizoplane soil's dominant fungi contained all of the rhizosphere soil's dominant fungi, indicating that the fungi in rhizoplane soils and rhizosphere soils are closely related. Within four periods, Mortierellaceae and Tricholomataceae were the dominant soil fungi in the flowering and fruiting rhizosphere soils, respectively. The seasonal variation of Hydnodontaceae was similar to that of Tricholomataceae, but this was not significant. Hypocreaceae and Mortierellaceae had the opposite seasonal variation as Tricholomataceae, with relative abundance increasing during flowering and decreasing during fruiting.

### Ecological functions of dominant fungi in microhabitats

According to the FungalTraits [37] database, Aspergillaceae are saprophytic fungi with no clear classification. Tulasnellaceae are orchid mycorrhizal fungi with litter saprophytism as their primary lifestyle, but they have a strong association with orchid roots [9]. Tricholomataceae are primarily soil saprophytes, and they are members of the CHEGD (the acronym of the constituent taxa: Clavariaceae, Hygrophoraceae, Entolomataceae, Geoglossaceae and Dermoloma) symbiotic fungi [38, 39]. The Hydnodontaceae are woody saprophytes [40]. Mortierellaceae have unspecified saprophytes and animal decomposers listed as their primary and secondary lifestyles [41]. Hypocreaceae are soil saprophytes [42]. It is possible that Helotiaceae are litter saprophytes. Litter saprophytism is the primary lifestyle of Trimorphomycetaceae, and animal decomposition is the secondary lifestyle. Bulleribasidiaceae are unspecified saprophytes, while Archaeorhizomycetaceae are soil saprophytes and orchid root-associated fungi with primary and secondary lifestyles. It can be seen that the primary lifestyles of the dominant fungal families are all saprophytic, whereas the dominant fungi in orchid roots and root surfaces are mostly symbiotic, and saprophytic ecotypes are mostly found in the dominant fungi in the rhizosphere soil.

### Characteristics of overall and interannual variation in microhabitat fungal diversity

**Fig. 2:**
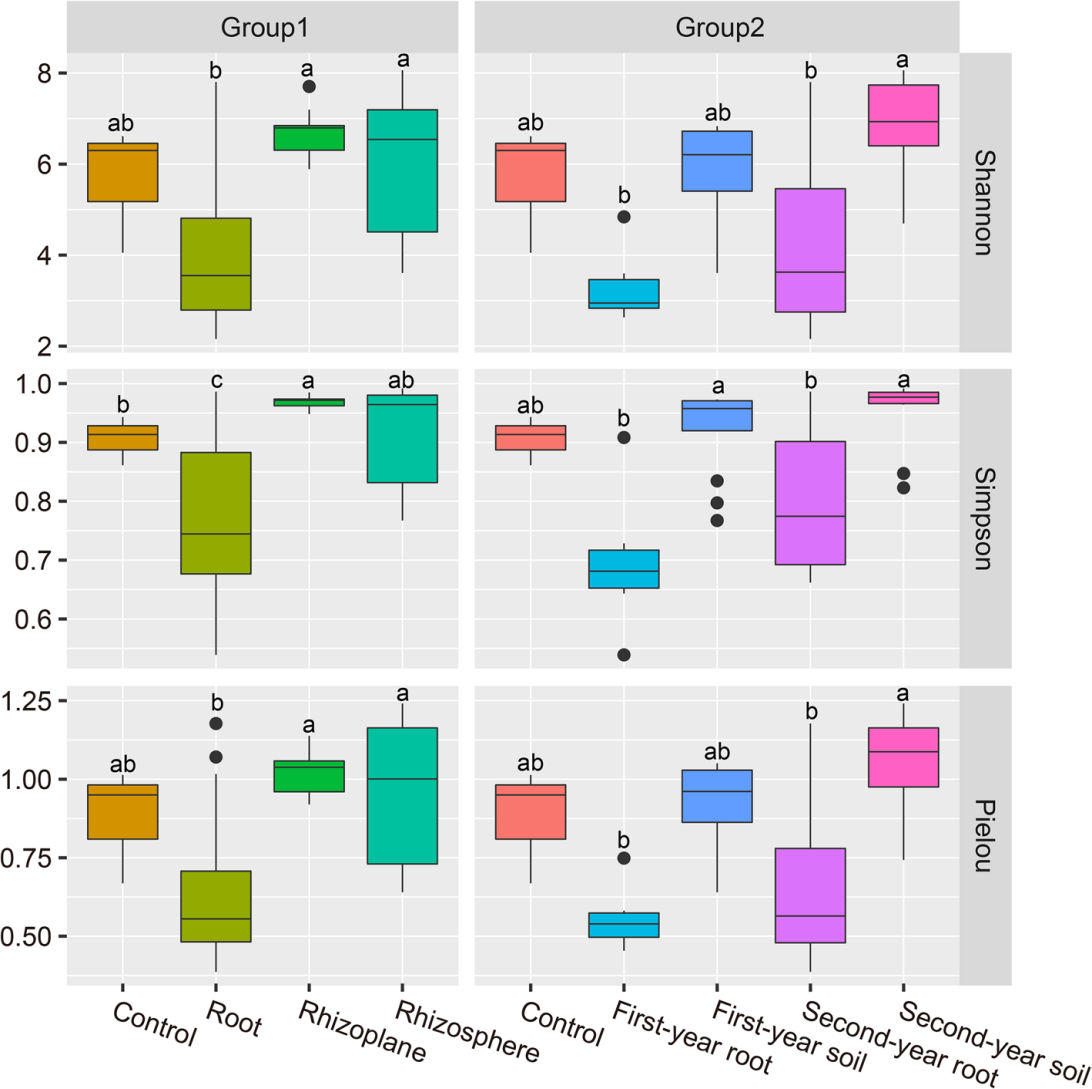
An analysis of alpha diversity indices for the overall and interannual fungal microhabitats of *C.sieboldii*. Group1 is divided into groups by spatial properties. Control, Root, Rhizoplane, and Rhizosphere indicate the blank control soil, orchid root, rhizoplane soil, and rhizosphere soil, respectively. Group2 is divided into groups by temporal properties. Control, First-year root, First-year soil, Second-year root, and Second-year soil indicate the blank control soil, plant roots of 2019, soil of 2019 (including rhizoplane soil and rhizosphere soil), plant roots for 2020 and 2021, and soils for 2020 and 2021, respectively. The ACE indices did not differ significantly between any of these subgroups, and thus they are not presented

The diversity of soil fungi and orchid endophytic fungi did not differ between years (Shannon, Simpson, Pielou, *P* < 0.05, Fig. 2). The diversity indices and evenness indices for the fungi in orchid roots and root-associated soil were significantly different (*P* < 0.05), and these indices were greater in soils than in orchid roots. This indicates that the abundance of fungal species in soils was much greater than in orchid roots and that the abundance of each fungal species in soils was more uniform, while dominant fungi with relatively high richness were found in the roots. Furthermore, there was a difference between the fungal species in the control soil and the rhizoplane soil according to the Simpson index. All of the diversity indices of the fungi showed little difference between rhizoplane and rhizosphere soils, but the range of diversity indices for rhizoplane soils appeared to be narrower than for rhizosphere soils, indicating that the fungal species composition of rhizoplane soils was stable and less variable.

### Seasonal variation characteristics of microhabitat fungal diversity

**Fig. 3:**
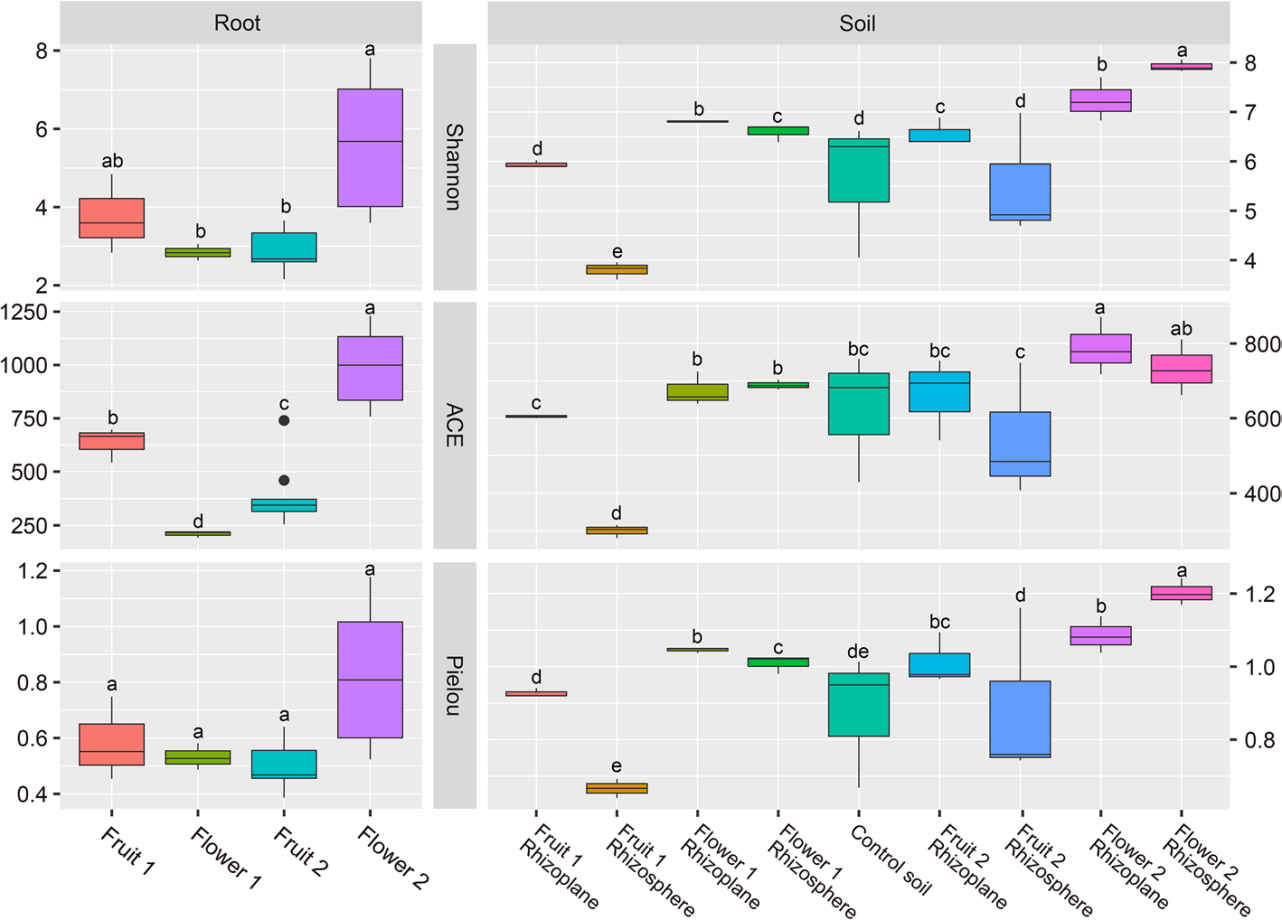
An analysis of alpha diversity in four phenological periods of the fungal microhabitats. Flower 1, Fruit 1, Flower 2, and Fruit 2 indicate the flowering of 2019, fruiting of 2019, flowering of 2021, and fruiting of 2020, respectively. The Simpson and ACE indices exhibit extremely similar patterns of variation, and therefore the Simpson indices have been omitted from this figure

Seasonal changes were observable in the fungal composition of the orchid roots (abundance-based coverage estimator [ACE], *P* < 0.05, Fig. 3). The ACE index values of endophytic fungi decreased in the order of flowering of 2021, fruiting of 2019, fruiting of 2020, and flowering of 2019 in orchid roots. The Shannon index of endophytic fungi varied by season, but only the last flowering period and the two previous phenological periods were significantly different (*P* < 0.05). In general, there was no significant difference in the alpha diversity indices of endophytic fungal communities between years, but there were significant differences between seasons, implying that *C. sieboldii*'*s* endophytic fungal communities are characterized by seasonal variation. We believe that this variation is most likely related to the orchid's phenology.

Control soils had a different fungal composition than the root-associated soils (Shannon, Pielou, *P* < 0.05, Fig. 3). More interestingly, rhizoplane soil harbored a larger diversity of fungi than rhizosphere soil (Shannon, Pielou, *P* < 0.05). In all of the seasons, the diversity index and evenness index of fungi in the rhizoplane soil were significantly higher than those in the rhizosphere soil (*P* < 0.05), except for the diversity index and evenness index of fungi in the flowering of 2021 that were significantly greater than those in the rhizoplane soil in the same period (*P* < 0.05). In conclusion, the alpha diversity indices of rhizoplane soil fungi and rhizosphere soil fungi in the flowering stage were relatively similar, whereas the differences in alpha diversity indices between rhizoplane soil fungi and rhizosphere soil fungi in the fruiting stage were more significant. In particular, the alpha diversity index of rhizoplane soil fungi in the fruiting of 2019 was significantly greater than that of rhizosphere soil fungi in the same period.

### Microhabitat fungal community diversity characteristics

**Fig. 4:**
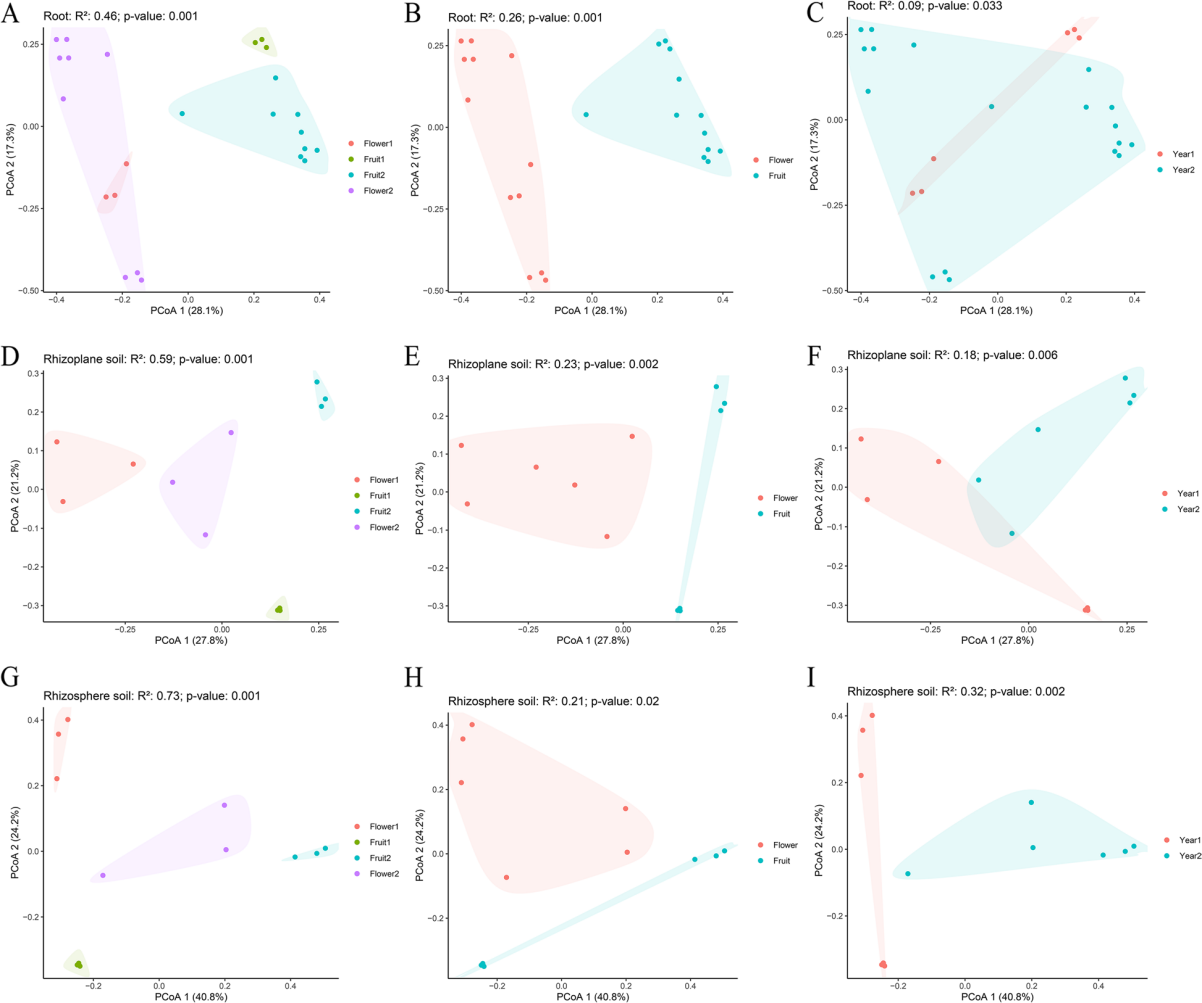
Spatial and temporal dynamic characteristics of the microhabitat community of this orchid by beta diversity analysis. **A-C**, **D-F**, and **G-I** indicate the temporal dynamics of fungal communities in orchid roots, rhizoplane soil, and rhizosphere soil, respectively. Flower 1, Fruit 1, Flower 2, and Fruit 2 indicate the flowering of 2019, fruiting of 2019, flowering of 2021, and fruiting of 2020, respectively. Flower, Fruit, Year 1, and Year 2 indicate the flowering, fruiting, 2019, and 2020 and 2021, respectively

The difference between fungal communities in rhizosphere soil was the largest in different periods, followed by rhizoplane soil and orchid roots (principal coordinates analysis [PCoA], Fig. 4). In other words, the closer the fungal community to the orchid's roots, the lower the variation, indicating a relatively stable state. More interestingly, the three spatial categories of fungi exhibited distinct temporal dynamic patterns: root and rhizoplane soil fungi demonstrated seasonal variation, whereas rhizosphere soil fungi displayed inter-annual variation (PERMANOVA analysis, Fig. 4). The R^2^ values of orchid root fungi decreased sequentially (the superscripts in Fig. 4A-C), indicating that grouping in each period could maximize the differences among different samples (*P* < 0.05). In addition, in rhizoplane soil fungi, the coefficients had the same characteristics as those mentioned above. The R^2^ values of each period in rhizoplane and rhizosphere soil were much greater than those for season and year (Fig. 4D-I), and the difference was significant (*P* < 0.05), indicating that this grouping could better illustrate the difference in fungal community structure than season or year. The rhizosphere soil of different periods had the most significant difference in fungal community structure (*R*^2^ = 0.73, *P* = 0.001). Year explained up to 32% of the rhizosphere soil fungi variation, whereas year explained only 9% of the total endophytic fungi variation (Fig. 4C, F, and I). The endophytic fungi and rhizoplane soil fungi in different years were similar, whereas endophytic fungi and rhizoplane soil fungi were separated from each other in different phenological periods (Fig. 4C and F), indicating that the seasonal differences in the structure of the fungal communities of orchid roots and rhizoplane soil were significantly greater than the interannual differences, while temporal dynamic features in rhizosphere soil fungi showed the opposite pattern. Finally, the interannual changes in fungal community structure in rhizosphere soil were more significant than the seasonal changes in roots and rhizoplane soil.

### Prediction of fungal functions in the microhabitat of this orchid based on the FungalTraits database

**Fig. 5:**
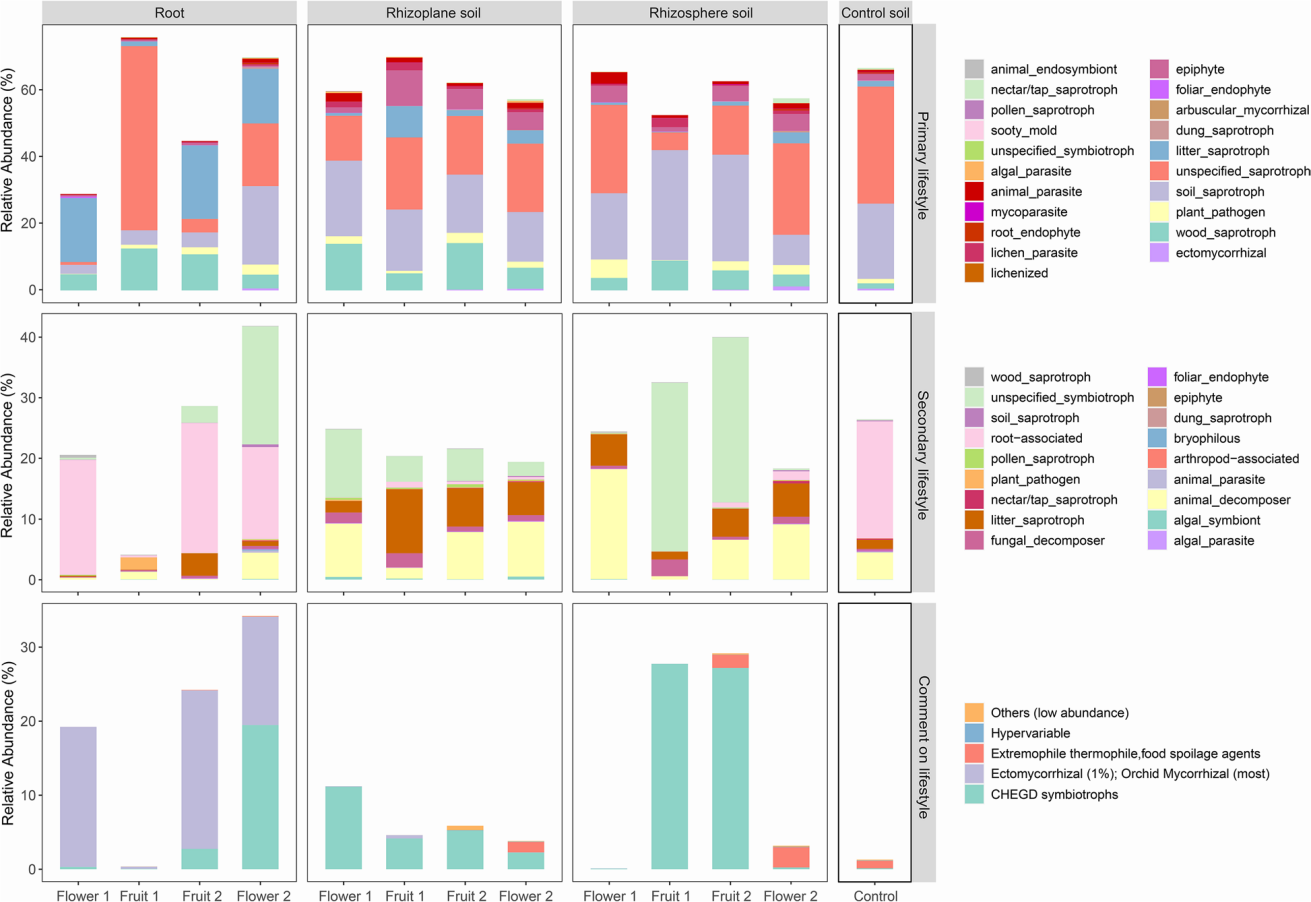
The relative abundance of fungal functions in terms of primary lifestyle, secondary lifestyle, and comments on lifestyle retrieved from the FungalTraits database. Flower 1, Fruit 1, Flower 2, and Fruit 2 indicate the flowering of 2019, fruiting of 2019, flowering of 2021, and fruiting of 2020, respectively

The FungalTraits database predicted the major ecological traits of 59.37% of the fungal sequences, while the FUNGuild [43] database annotated the ecological functions of 45.55% of the fungal sequences. Clearly, the FungalTraits database offered more data, and thus we chose it to display the functional characteristics of the fungal community in this orchid's microhabitat. The ecological traits of endophytic fungi associated with *C. sieboldii* were mostly symbiotic (e.g., orchid mycorrhizal fungi), and fungi in the root-associated soil were dominated by saprophytic and symbiotic types, whereas saprophytic fungi dominated the control soil (Fig. 5). There was a clear distinction in fungal ecotypes between orchid roots and soil in the classification of primary lifestyle: in orchid roots, the fungal community was dominated by unspecified saprophytes, litter saprophytes, soil saprophytes, and wood saprophytes; in root-associated soil, the fungal community was dominated by unspecified saprophytes, soil saprophytes, and wood saprophytes. Unspecified saprophytic and woody saprophytic fungi dominated control soil. Orchid roots were dominated by unspecified symbiotic fungi and root-associated fungi in their secondary lifestyle; rhizoplane soils were dominated by unspecified symbiotic fungi, litter saprophytic fungi, and decomposing animal decomposer fungi in their secondary lifestyle, and the main ecological types of rhizosphere soils were similar to those of rhizoplane soils. The relative abundances of undefined symbiotic fungi and animal decomposer fungi were much higher in rhizosphere soils than in rhizoplane soils; soil fungi in the control group were dominated by animal decomposing fungi and plant root-associated fungi. The ecological traits of fungi in orchid roots appeared to differ from those in the soil in the comments on lifestyle in the database. The majority of fungi in roots were orchid mycorrhizal fungi (Tulasnellaceae), and another fraction was CHEGD symbiotrophs. The fungi of this ecotype also occupied a high proportion of the rhizoplane and rhizosphere soils, whereas the control soils only had fungi of more thermophilic and saprophytic types (extremophile thermophile and food spoilage agents). The results clearly show that *C. sieboldii*'s microhabitat had a higher concentration of symbiotic fungi near the orchids.

## Discussion

In this study, we found three families of dominant fungi in orchid roots, seven families of dominant fungi in rhizoplane soil, and four families of dominant fungi in rhizosphere soil. The host orchid can allow two or more species of fungi to colonize the roots [20, 44–46], similar to our findings. The number of dominant fungal families was greater in rhizoplane soil than that in rhizosphere soil, and both rhizoplane soil and rhizosphere soil shared all of the dominant fungal families. On the one hand, not all fungi can colonize orchid roots as endophytic fungi do, resulting in a lower number of dominant fungal families in orchid roots than in rhizoplane soil. Orchid roots, on the other hand, may release substances that attract fungi to congregate on the root surface [47], resulting in more dominant fungal families in rhizoplane soils than in roots or rhizosphere soils. Furthermore, there were differences in dominant fungi and diversity between the control soil and the root-associated soil, implying that orchids could maintain or regulate the fungal community structure in the soil near the roots by selectively enriching useful fungi and thereby increasing the abundance of symbiotic fungi closer to the orchid. It remains unclear how exactly orchids control fungi, and thus further research is needed in this area.

Several endophytic fungi can exist in orchids simultaneously during certain periods, while a specific type of fungus may be dominant during other periods. This may be due to the fact that orchids have different needs during different phenological stages and choose to live in association with fungi that serve different ecological functions at different times. Some orchid species express high fidelity towards specific fungi [48]. This is similar to the situation observed in the first three stages; *C. sieboldii* is only associated with a specific fungal family in each stage. However, in the flowering of 2021, there were three dominant fungal families in the orchid roots. Preferential effects and bidirectional selective pressures between fungi and their hosts could contribute to the replacement of fungal species with time [49]. Accordingly, we hypothesized that orchids should choose fungi that are most beneficial or those fungi that have the best colonization ability to stay within the orchid roots. Interestingly, the endophytic fungi of *C. sieboldii* had the same dominant taxa in 50% of the sampled periods, implying that the corresponding optimal fungi colonized the orchid roots repeatedly during each reproductive season, possibly as a result of the previously mentioned bidirectional selection of fungi and orchids. Furthermore, because the seasonal variation in endophytic fungal community structure was greater than the interannual variation, we hypothesized that orchid phenology was one of the key factors influencing endophytic fungi colonization. However, further experiments are needed to investigate the mechanism of this effect. According to some studies, the mycorrhizal fungal infection reaches its peak intensity after 2–6 months of development, and the pelotons degrade regularly [10, 47, 50]. These findings suggest that endophytic fungi colonization was dynamic, a conclusion that supports our point about the periodic change in the abundance of dominant endophytic fungi.

Tulasnellaceae is a dominant endophytic fungal family that appears most frequently in the roots of orchids, but whether it has a seasonal preference has been a point of contention. Tulasnellaceae has also been reported to be more common in summer, autumn and winter among other orchids [22, 24]. In this study, Tulasnellaceae may have preferred the flowering stage before randomly appearing in the orchid's roots during the fruiting stage. In addition, Tulasnellaceae as Rhizoctonia fungi is a common fungal taxon of orchid mycorrhizal fungi [51]. Therefore, we consider Tulasnellaceae to be the putative mycorrhizal fungi of *C. sieboldii*. Aspergillaceae was a dominant endophytic fungal family in two phenological periods, and its main lifestyle was saprophytic according to the FungalTraits database. However, Aspergillaceae (*Penicillium* and *Aspergillus*) have already been recorded as Orchid mycorrhizal fungi by Wang et al. [52]. Furthermore, because the relative abundances of *Penicillium* and *Aspergillus* were relatively high in this study, we believe that Aspergillaceae belongs to *C. sieboldii*'s guild of mycorrhizal fungi. Some saprophytic and ectomycorrhizal (ECM) fungi have recently been discovered to form symbiotic relationships with orchids [51, 53, 54]. These findings suggest that fungi that live in symbiotic relationships with orchids may have a complex life cycle, living in different parts in or out of orchids at different times and in different ways. Endophytic and saprophytic fungi have similar phylogenetic origins, and endophytic fungi have the ability to switch ecological strategies to become saprophytic during host senescence [55]. We hypothesize that endophytic fungi are mutually beneficial to orchids during their healthy growth periods, and they turn to decomposing orchid tissues to live during the orchids' latter stages of the life cycle. Following this thought, Aspergillaceea may have different ecological strategies at different times. The triggering mechanism for these two ecological state transitions needs to be further investigated and verified. Tricholomataceae, the dominant fungi in all three types of samples, has saprophytic and symbiotic ecological types. Therefore, we hypothesize that some fungal taxa under this fungal family most likely act as a “pipe” for nutrient transfer and exchange between plant roots and the surrounding soil. These fungi may form a mycelium network: the saprophytic fungi are responsible for decomposing humus in the soil to obtain nutrients; the symbiotic fungi deliver nutrients to orchid roots via the mycelium “network” in exchange for other necessary resources for survival, resulting in a “trade” symbiosis with orchids. The dominant fungi, Tricholomataceae, are abundant in the roots and surrounding soils of pine trees [49]. Furthermore, saprophytes are closely associated with some orchids and form root-like mycelia that connect orchid roots to litter, thereby allowing important material elements such as carbon and nitrogen to be transferred from litter to orchids [56–59].

The rhizosphere soil's fungal community was the most variable, followed by the rhizoplane soil, and the fungal community in the orchid roots was the least variable. We believe that this is because rhizosphere soils are located far from the orchid and are more susceptible to large changes in fungal communities as a result of external environmental factors such as temperature and precipitation [28, 60]. In addition, fungi in orchid roots live in a relatively enclosed space and are more influenced by the orchid's internal physiology and ecology. More importantly, the host orchid to a large degree determines which fungi are allowed to colonize the root [61], and thus the endophytic fungal communities are less variable. Because it is closer to the orchid roots, the rhizoplane soil can be influenced by the orchid's rhizoplane secretions, resulting in a less variable fungal community. In general, the closer one gets to the orchids, the more likely it is that the fungal community will be stabilized by orchid influence.

The diversity and community structure in orchid roots and rhizoplane soil did not differ significantly from year to year, but it did differ significantly from season to season. There has been debate in recent years about whether endophytic fungal communities have clear temporal dynamics. Orchid phenology influences the colonization of Orchidaceae mycorrhizal fungi, leading to seasonal changes [21, 24]. Richard et al. (2011) discovered an ECM community that had remained stable for at least two years [28]. Furthermore, because studies of seasonal changes in endophytic fungal communities typically only consider sequential sampling for a few months of the year [26, 27, 29, 60], we were unable to determine whether the dynamics of these fungal communities truly reflected seasonal changes or simply reflected the fact that the fungal community was changing at a certain rate. Microbial communities, for example, change at a rate of 0.010–0.025 per year, with fungi changing slightly faster than bacteria in the soil, about 2.5 times faster than soil bacteria [62]. Our results do not demonstrate a seasonal preference for high fungal diversity; our results simply reflect that the 2019 fruiting and 2021 flowering periods had greater fungal diversity than other periods. In sum, our results suggest that the fungal community of the *C. sieboldii* microhabitat changes at a certain rate each year, but the intra-annual variation is somewhat greater than the interannual variation. Nonetheless, these findings may be limited by the size of the dataset. Given the high spatial heterogeneity of the soil microbiota [63] and one study that found differences in the percentages of mycorrhizal formation among different individuals of the same orchid species [64], we recognize that our use of a limited number of samples resulted in a relatively small dataset, and this limits the strength of the conclusions. However, we believe that the results of this exploratory study are still relevant, as the data provide reference information on the temporal dynamic patterns of fungal communities at a small spatial scale (1 cm level).

Both unspecified saprophytes and wood saprophytes were the primary lifestyles in all of the samples in terms of nutrient types, whereas the saprophytic lifestyles of fungi closer to orchid roots were more complex, and more symbiotic fungi were closer to orchids. *C. sieboldii* prefers shady and wet habitats as an understory plant, and the majority of their distribution sites are at the feet of shady slopes near streams and are covered in a layer of litter. Thus, saprophytes met our expectations as the dominant ecological functional group in *C. sieboldii's* microhabitat. This was supported by the fact that saprophytic fungi were the most common endophytic fungi in other orchid roots (50.11%–85.98%) [65]. As mentioned earlier, seeds of orchids can only germinate efficiently if they are colonized with the appropriate symbiotic fungi [17, 48]. In situ germination experiments revealed that the germination rate of orchids decreased rapidly as the distance between the seeds and the nearest aboveground plant increased [66, 67]. This is in line with our findings that the closer the sites were to the orchid, the more symbiotic fungi were present. However, the experimental approach of using two primers may have distorted these comparative results regarding the three spatial classes of fungal communities. Different primers have preferences for various taxa, and this may introduce systematic bias into the results [68, 69]. This limitation implies that results concerning comparisons between plant roots and soil need to be interpreted cautiously.

## Conclusion

Fungi are unquestionably important to orchids, and here we present the characteristics of microhabitat fungal communities associated with *C. sieboldii*. The orchid roots, rhizoplane soil, and rhizosphere soil all had similar but not identical dominant fungi in *C. sieboldii*'*s* root microhabitats. At the same time, the closer the fungi were to the orchid roots, the more symbiotic fungi were present. More interestingly, the fungal communities closer to the orchid roots displayed seasonal variation, whereas the interannual variation of the fungal communities farther away from the orchid roots was more noticeable. We believe that this seasonal variation is due to the species' ability to self-balance and adjust at each life stage in order to make the best use of available resources. Since root fungal communities varied seasonally instead of annually, we suggest that in similar research conducted in orchids' microhabitat, sampling at different times of the year is necessary to reduce chance error. Given that the scope of our current study is limited to the state of fungal communities at a single location and in the same habitat, it is impossible to assess the applicability of such dynamic patterns on a larger scale, and in-depth studies at multiple sites with the same species over a longer period of time may be required. Furthermore, using a series of inoculation tests to examine the growth effects of various dominant fungi on orchids and their functional traits would be an important step toward understanding orchid conservation and expansion.

## Materials and methods

### Sampling of plants and soil materials

All of the samples were collected in a way that caused as little damage to the habitat as possible and without putting *C. sieboldii*'s survival at risk [70]. Samples of roots, rhizosphere soil, and rhizoplane soil were collected from the same spot in Jing County, Xuancheng City, Anhui Province, China, in April 2019, September 2019, September 2020, and April 2021. This is where the largest wild population of *C. sieboldii* is currently found. One year is defined as one complete flowering and fruiting cycle of *C. sieboldii*. For the subsequent analysis, we assigned all of the samples collected in April (flowering) and September (fruiting) of 2019 to the first year, and samples collected in September (fruiting) of 2020 and April (flowering) of 2021 to the second year. Because the population of this orchid is so small, root samples were collected from three distinct plants during each period. The distance between these orchids was very small (less than 0.5 m). Three roots were sampled from each orchid plant as one sample, for a total of 12 root samples. A total of 24 soil samples were collected, three for each kind of soil in each period. To protect the population, the number of replications was limited, and this limited the statistical analysis. In April 2021, three soil samples were taken from three locations five meters apart from the same population as control soil. The Department of Wildlife Conservation and Nature Reserve Management of the National Forestry and Grassland Administration of China is the highest administrative agency for wildlife management in China. Our research was conducted under a project commissioned by the department and permission to collect plant material was included in this project. At the time the plant material was collected (before April 2021), the orchid was not a protected species in China [3]. Our collection was therefore lawful and compliant, and no additional permission was required to collect the plant material. The plant material was identified by Shaohua Xing, the first researcher to study *C. sieboldii* in mainland China [6]. Based on the available research and a photograph of a type of the orchid specimen from the China Plant Science Data Center (CSFI029077, preserved in the Forest Herbarium, College of Forestry, Central South University of Forestry and Technology), Shaohua Xing confirmed that the plant material was from *C. sieboldii*. To protect the plant population, we did not collect complete plants as identification voucher specimens. The Wild Plants Protection Regulation of China was complied with in the collection and treatment of the materials for this experiment. Our plant material was collected using non-lethal, minimum quantity collection principles that fully adhered to the IUCN's "Policy Statement on Research Involving Species at Risk of Extinction," with Special Reference to Scientific Collecting of Threatened Species, Implementation Guidelines (v1.0).

**Fig. 6:**
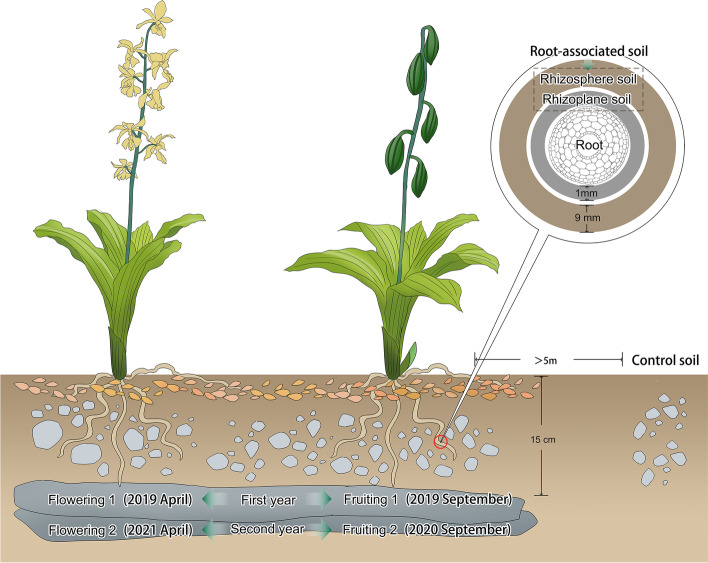
Sampling strategy diagram. Twenty-two individuals of *C.sieboldii* were grown on the same rock mass. Samples were taken at this site for all four phenological periods (2019 flowering, 2019 fruiting, 2020 fruiting and 2021 flowering). Three spatial categories were delineated (root, rhizoplane, and rhizosphere)

The habitat in which *C. sieboldii* grows has a thin soil layer of about 10–15 cm thickness. The soil profile (from the soil surface to the rock surface) was dug, and three intact plant roots were taken from each orchid. The dead material and large clumps of soil on the root surface were removed; the roots were placed in a 10 ml plastic centrifuge tube and transported in dry ice buckets. Rhizosphere soil was defined as soil that was 0.01–1 cm away from the root surface and was shaken off and stored in one 5 ml plastic centrifuge tube after sieving through a 2 mm soil standard sieve. Rhizoplane soil was defined as soil within 0.1 mm of the root surface, and soil particles were removed from the root surface using a NT-285 ultrasonic cleaning instrument (Guangzhou Hengwei Electronics Technology Co., Ltd., China) and then were pooled into one 5 ml plastic centrifuge tube. The rhizosphere soil and rhizoplane soil were considered the root-associated soil (Fig. 6). Each plant's soil was sampled as much as possible to ensure that > 5 g of soil was obtained per sample. Due to the proximity of the orchids to the cliff (south) at the study site, we collected one control soil sample from each of the three directions of the orchid samples (north, east, and west). In addition, habitats similar to the study site had to be chosen, and control soils had to be collected at a distance of at least 5 m from the study site. Dead material was removed from the soil surface; soil profiles were excavated; one portion of the soil was taken from each of the profile's upper, middle, and lower layers and thoroughly mixed, and then sieved with 2 mm soil standard sieves and stored in a 5 ml plastic centrifuge tube. Orchid roots that were fresh and healthy were chosen. The roots were rinsed with distilled water, cut into small sections that were 2–3 cm in length, washed with distilled water, soaked in 0.1% HgCl_2_ for 5–8 min, removed and rinsed with distilled water once or twice, then soaked in 75% alcohol for about 20 s, dried, and placed in 5 ml plastic centrifuge tubes. To keep the samples stable, they were moved to a –80 °C refrigerator after they were cleaned.

### PCR amplification of root and soil samples

The orchid root samples and soil samples were used to extract DNA using a NucleoSpin 96 Soil kit (Macherey–Nagel, Germany) after grinding. The primer pair ITS1-5F (ITS5: 5'-GGAAGTAAAAGTCGTAACAAGG; ITS2: 5'-GCTGCGTTCTTCATCGATGC-3') amplified the ITS1 region of the fungal genome in the orchid tissue samples [71]. The primers ITS1F (5'-CTTGGTCATTTAGAGGAAGTAA-3') and ITS2 (5'-GCTGCGTTCTTCATCGATGC-3') were used to amplify the ITS1 region of the fungal genome in the soil samples [72]. Biomarker Technologies (Biomarker, Beijing, China) performed primer synthesis and sequencing. The PCR reaction system contained 10 μL and comprised 1 μL DNA template, 3 μM upstream primers, 3 μM downstream primers, 5 μL KOD FX Neo Buffer, 4 mM dNTP, and 0.2 μL KOD FX Neo supplemented by ddH_2_O to 10 μL. The PCR amplification process was as follows: pre-denaturation at 95 °C for 5 min, denaturation at 95 °C for 30 s, annealing at 50 °C for 30 s, extension at 72 °C for 40 s, and 35 cycles. After being expanded at 72 °C for seven minutes, it was kept at 4 °C. Then, the products of PCR were analyzed using agarose gel electrophoresis. Electrophoresis was performed for 40 min at 120 V using 1.8% agarose gel. The product was blended according to the mass ratio of 1:1 based on the electrophoresis quantitative data. An OMEGA Cycle Pure Kit (Omega Bio-Tek, USA) was used to purify the mixed products. After 40 min of electrophoresis at 120 V voltage in 1.8% agarose gel, the target fragments were cut and recovered using a Monarch DNA Gel Extraction Kit (New England Biolabs, USA). An Illumina Hiseq2500 PE250 platform was used for bidirectional sequencing.

### Bioinformatic and statistical analyses

The raw data from sequencing were quantified by using the microbial ecology bioinformatics software QIIME2 (v2021.4) in the VM Oracle VirtualBox (v6.1.30) [73]. After removing the primers, the sequences were subjected to a noise reduction process using the DADA2 plugin, and representative sequences and feature tables were created. We used Amplicon Sequence Variants (ASVs) instead of Operational Taxonomic Units (OTUs) to reduce potential sequencing errors and to make the data more realistically reflect the actual level of fungal diversity [74]. The vsearch plugin then clustered the feature table at a 99% similarity level to obtain ASVs. At a confidence level of 0.8, the representative sequences obtained from clustering were annotated using the taxonomic database UNITE (v8.3) [75]. Because the number of sequences obtained from sequencing was not uniform, we used the median of read numbers of most of the samples as the threshold value for data sampling flatness, and the vegan package of R (v4.1.1) randomly selected 80,000 sequences for samples with greater than 80,000 read numbers, while samples with less than 80,000 read numbers were retained. ASVs that occurred ≤ three times were removed from the results, as these were likely to be errors.

FUNGuild, the most widely used fungal functional database, has 9476 entries with a 66% genus-level identification range and a 34% species-level identification range [43]. Põlme et al. recently developed the FungalTraits tool that was used to re-annotate 10,210 fungal genera and 151 Stramenopila genera associated with 17 lifestyles [37] based on the previous fungal functional annotation tools FUNGuild and Fun^Fun^ [76]. To reveal the functional characteristics of the fungal community in the microhabitat of *C. sieboldii*, we downloaded the python package from GitHub (https://github.com/UMNFuN/FUNGuild) and performed fungal function prediction based on the FUNGuild database (v.1.1) using the Ubuntu (v18.04) system. All of the fungal functional data information tables were obtained from the appendices of research papers in the publication database. We then assigned fungal trait information from the FungalTraits database (v.1.2) to the dataset generated from this experiment using Excel's vlookup function. We found that the FUNGuild and FungalTraits databases both explained the sequence data of soil fungi and orchid endophytic fungi considered in this paper to a similar extent, but the FungalTraits database had the most efficient annotation. Therefore, to test the hypothesis that different spatial categories possess different functional types of fungi, different functional groups of fungi in orchid microhabitats were assigned based on the FungalTraits database. Finally, we used R's reshape2 and ggplot2 packages to create histograms of the relative abundance of fungal community functions in each spatial category.

The dominant fungi were classified in each sample to allow a more visual comparison of fungal community distinction across the three spatial categories and two phenological stages. The two most popular methods for classifying dominant taxa are maximum relative abundance [77–79] and relative abundance > 1% [80, 81]. If the dominant fungi are designated using the method of maximum relative abundance, it may result in the omission of other essential fungi. Similarly, if dominant fungi were defined by relative abundance > 1%, there would be too many dominant fungi, resulting in information redundancy and confusion. To determine the dominant fungi in each sample, we therefore utilized the minimum value of the maximum relative abundance as the critical value. This method would permit certain samples to contain two or more dominant fungi. To determine which dominant fungi were the key fungi affecting the growth of orchids, we further elucidated the different ecological functions of these dominant fungi based on the FungalTraits database.

The spatial distribution patterns and temporal dynamics of fungal communities in orchid microhabitats were investigated using alpha and beta diversity analyses from the perspectives of individual samples and between samples. The Shannon and Simpson diversity indices, the ACE richness index, and the Pielou evenness index were all calculated in R using the diversity() function of the vegan package. The Shannon and Simpson diversity indices consider both species richness and evenness [82, 83]. The Shannon diversity index takes more species with low relative abundance into account, whereas the Simpson diversity index takes more species with high relative abundance into account. The ACE richness index estimates the number of species in a community that have not yet been observed to determine the number of species in a single sample [84]. The Pielou evenness index was used to measure the relative species richness in a single sample [85]. The four alpha diversity indices were used to quantify fungal diversity in a single sample as well as to compare similarities and differences between samples. The above diversity indices were nonparametrically tested using the reshape2 and npmc packages via Kruskal–Wallis tests to compare the differences between the overall samples, and the significance "abc" label was determined based on the highest median and the *P*-value: the group with the highest median was labeled as "a." If the group with the highest median was not significantly different from the second-highest group, the group with the second-highest median was not significantly different from the third-highest group, and the group with the highest median was significantly different from the third-highest group, then the second-highest group was labeled as "ab," the third-highest group was labeled as "b," and so on. Beta diversity analysis was carried out using R's vegan and ggalt packages, including principal coordinate analysis (PCoA) [86] and permutational multivariate analysis of variance (PERMANOVA) [87]. ASVs data of each sample were visualized using PCoA with dimensionality reduction based on the Bray–Curtis distance matrix. To compare the fungal community structures of root, rhizoplane, and rhizosphere samples within three temporal groups, samples were grouped based on temporal attributes. Based on the results of PCoA, which showed that some samples from different groups were overlapping, PERMANOVA (Adonis, 999 permutations) was used to test for significant differences in the structure of the fungal communities between these groups. The ggplot2 package was used to plot the results of the above analyses.

## Data Availability

Raw amplicon sequence data related to this study were deposited in the NCBI Sequence Read Archive (NCBI SRA) under Bioproject PRJNA852404. Given that the study's subject (*C. sieboldii*) belongs to small population species in the IUCN standard, we collected a small sample size and used all samples for experiments, leaving no plants behind.

## Abbreviations

ACE: Abundance-based coverage estimator
AMF: Arbuscular mycorrhizal fungi
ANOVA: Analysis of variance
ASV: Amplicon sequence variant
CHEGD: the acronym of the constituent taxa: Clavariaceae, Hygrophoraceae, Entolomataceae, Geoglossaceae and Dermoloma
ECM: Ectomycorrhizal
ITS: Internal transcribed spacer
OTUs: Operational taxonomic units
OMF: Orchid mycorrhizal fungi
ONF: Orchid non-mycorrhizal fungi
PCoA: Principal coordinate analysis
PERMANOVA: Permutational multivariate analysis of variance
